# Supplementary material for: Microplastic-Contaminated Feed Interferes with Antioxidant Enzyme and Lysozyme Gene Expression of Pacific White Shrimp (Litopenaeus vannamei) Leading to Hepatopancreas Damage and Increased Mortality
Source: Animals (Basel). 2022 Nov 26;12(23):3308. doi: 10.3390/ani12233308 (PMC9740652; doi:10.3390/ani12233308)
Supplement: Supplementary file 1 [file animals-12-03308-s001.zip › animals-1973387-supplementary.pdf]

Microplastics-contaminated Feed Interferes with Antioxidant and Lysozyme Gene Expression of Pacific white shrimp (*Litopenaeus vannamei*) leading to Hepatopancreas Damage and Increased Mortality

Supplementary Material

Table S1. HDPE content in feed used in the toxicity test.

Type of feed	Grams of HDPE-MP per kilogram of feed	Purpose
0%	0	Negative control
0.5%	5	Test feeding
5%	50	Test feeding
10%	100	Test feeding
20%	200	Test feeding

Table S2. HDPE content in feed used to determine microplastic effects on gene expression and histopathology.

Type of feed	Grams of HDPE-MP per kilogram of feed	Purpose
0%	0	Negative control
0.1%	1	Test feeding
0.5%	5	Test feeding
1%	10	Test feeding
3%	30	Test feeding at LD_{50} dosage

Table S3. Nutrient composition of base shrimp feed

Nutrient	Portion of nutrient
Protein	>40%
Lipid	>6%
Moisture	<10%
Fiber	<3%

Table S4. Details of temperature recirculation settings for qPCR

Step	Time	Temperature	Number of cycles
PCR initial activation	2 min	95°C	1
**3 step cycling**			
Denaturation	10 sec	95°C	45
Annealing	30 sec	60°C	
Extension	30 sec	72°C	

Table S5. Number of shrimp with different histopathological score for each lesion (n for each treatment = 6). Score 0 = No histopathological lesion in any field on the slides; 1 = Histopathological lesion present in <25% of the fields on the

slides; 2 = Histopathological lesion present between 25% to 50% of the fields on the slides; 3 = Histopathological lesion present

Dosage	Score	Number of shrimp with histopathological lesion (n=6)				
		Intersitial hemocyte infilltration	Epithelium hyperplasia	Tubular deformity	Nodule formation	Melani-zation
0%	0	6	6	6	6	6
	1	0	0	0	0	0
	2	0	0	0	0	0
	3	0	0	0	0	0
	4	0	0	0	0	0
0.1%	0	0	1	0	2	3
	1	3	2	1	2	3
	2	2	2	0	1	0
	3	0	0	2	0	0
	4	1	1	3	1	0
0.5%	0	1	2	0	1	3
	1	1	3	0	1	2
	2	2	1	1	3	1
	3	1	0	0	0	0
	4	1	0	5	1	0
1%	0	0	3	0	1	2
	1	2	2	0	1	3
	2	2	1	0	3	0
	3	2	0	2	0	1
	4	0	0	4	1	0
3%	0	0	5	0	0	0
	1	0	1	0	0	1
	2	1	0	0	1	2
	3	1	0	0	4	2
	4	4	0	6	1	1

Table S6. The mortality rate of shrimp during the second phase of the experiment on day 28 (The sacrificed shrimp before day 28 were not included)

Dosage	Mortality rate
Control (0%)	0%
0.1%	19.05%
0.5%	23.81%
1%	42.86%
3%	47.62%
